# Western Blot-Based Logistic Regression Model for the Identification of Recent HIV-1 Infection: A Promising HIV-1 Surveillance Approach for Resource-Limited Regions

**DOI:** 10.1155/2018/4390318

**Published:** 2018-01-14

**Authors:** Jiegang Huang, Minlian Wang, Chunyuan Huang, Bingyu Liang, Junjun Jiang, Chuanyi Ning, Ning Zang, Hui Chen, Jie Liu, Rongfeng Chen, Yanyan Liao, Li Ye, Hao Liang

**Affiliations:** ^1^Guangxi Key Laboratory of AIDS Prevention and Treatment and Guangxi Universities Key Laboratory of Prevention and Control of Highly Prevalent Disease, School of Public Health, Guangxi Medical University, Nanning, Guangxi, China; ^2^Guangxi Collaborative Innovation Center for Biomedicine, Life Science Institute, Guangxi Medical University, Nanning, Guangxi, China; ^3^Geriatrics Digestion Department of Internal Medicine, The First Affiliated Hospital of Guangxi Medical University, Nanning, Guangxi, China

## Abstract

**Objectives:**

Identifying recent infections is necessary to monitor HIV/AIDS epidemic; however, it needs to be further developed.

**Methods and Results:**

Participants were defined as having recent infection or older infection according to the estimated duration of HIV-1 infection and further assigned into training set and validation set according to their entering time points. Western blot (WB) confirmatory test and BED-CEIA were performed. The performance of the two methods on recent HIV-1 diagnosis was evaluated and compared. 81 subjects were enrolled in the training set and 72 in the validation set. Relative grey ratios of p24, p39, p31, p66, gp41, and gp160 were significantly higher in older infected patients of the training set. The present status of p55 was more frequently missing in recently infected patients in both sets. The logistic stepwise regression analysis of WB method shows sensitivity, specificity, and accuracy of 93.02%, 92.11%, and 92.59%. For BED-CEIA, they were 76.74%, 86.84%, and 81.48%. In the validation set, overall agreement rate, sensitivity, and specificity were 88.46%, 84.78%, and 86.11% in the WB-based method and 50.00%, 84.78%, and 72.22% in the BED-CEIA method.

**Conclusions:**

WB-based method is a promising approach to predict recent HIV-1 infection, especially in resource-limited regions.

## 1. Introduction

The prevalence of HIV-1 infection remains a serious concern, although some improvement in control and prevention of it has been achieved. According to the World Health Organization (WHO), of the 36.9 million people currently infected with HIV-1, two million are cases of recent infection [1]. According to the Chinese Centre for Disease Control and Prevention, 50,330 HIV-1 infections were reported in 2015. Therefore, identifying recent HIV-1 infections from the newly reported cases is critical for the accurate estimation of HIV-1 epidemic. In China, recent HIV infections are defined as those infected within 12 months. This duration is significant to HIV surveillance, especially incidence surveillance. In practice, half a year is usually set as the follow-up interval, and incidence is generally calculated annually [2, 3].

BED-CEIA is the most commonly used method for the identification of recent infections within 153 days (recency period). However, it is affected by CD4^+^ T cell counts, HIV-1 viral loads, and antiretroviral treatment (ART), as well as territory and HIV subtype [4–6]. Therefore, WHO did not recommend using BED-CEIA as a tool of recent incidence estimation and epidemiologic surveillance of HIV-1 [7]. Recently, line immunoassay (INNO-LIA HIV I/II Score assay) has been used to establish a method for recent HIV-1 infection diagnosis. However, the sensitivity of this algorithm is extremely low (59.4%) [8]. More recently, the Recent Infection Testing Algorithm was developed. Meanwhile, compared with these algorithms, multiassay algorithms are currently the most popular. Its performance was validated in populations of men who have sex with men, intravenous drug users, and female-infected cohort. However, its complicated procedure and requirement of accurate CD4^+^ T cell counts and/or viral load impeded its application in resource-limited countries. Although these methods showed some degree of sensitivity and specificity, applying them as routine surveillance tools is not feasible because of their false-positive rates and/or complicated procedures.

Western blot (WB) immunoassay was developed by Schupbach et al. in 1984 [9] to confirm HIV infection [10] and is one of the algorithms that detect antibodies of envelope glycoproteins (gp41, gp120, and gp160),* pol* proteins (p51, p31, and p66), and* gag* proteins (p17, p24, and p39) in a single test. Variations in WB band numbers, intensities, and order of appearance during different stages of HIV infection have been observed [11, 12].

Despite the availability of fourth-generation assays and automated commercial assays for HIV confirmation and recent HIV infection detection [13], they remain inaccessible in some countries, including China, because of patent protection policy and their extremely high prices. These countries or regions have accumulated a large number of WB strip test results in third-generation assays. If their information can be used for the estimation of newly reported HIV infections and AIDS epidemic, that will be significantly helpful for the control of AIDS in these countries. Previous studies were able to identify recent HIV infections on the basis of the presence of WB bands, but these approaches had extremely low sensitivity and specificity and thus are infeasible in reality [14].

In the current study, we intended to develop a practical, accurate, and cost-effective strategy for recent HIV-1 diagnosis and routine monitoring, especially in resource-limited settings. In view of potential of WB strips in differentiating recent HIV infection, we attempted to establish a quantitative measurement method and develop a simple but precise method for classifying recent HIV-1 infection on the basis of antibody reactivity in WB profiles.

## 2. Methods

### 2.1. Study Design and Patients

This study was part of the National Key Science and Technology Project of China. The samples used in this study were collected from four sites of Disease Control and Prevention Centres in Guangxi, China, including those in Liuzhou, Guigang, Chongzuo, and Qinzhou cities between August 2014 and December 2015, and were regularly tested for HIV infection every 3 months. Patients were assigned to training or validation sets according to their enrolment time points. Individuals with estimated duration of infection (EDI) of less than 1 year were classified as recent infection, whereas those with EDI longer than 1 year were classified as older infection [2]. The estimated date of infection was determined by the midpoint between the last HIV-negative test result and first HIV positive test result [15]. EDI was calculated from the estimated date of infection to the date of sampling. HIV infections of participants were screened by a third-generation enzyme-linked immunosorbent assay (Beijing Modern Gaoda Biotechnology Co., Beijing, China) and confirmed by WB test. For the prevention of influence of other clinical conditions on HIV immune status, all participants included had no fatal chronic diseases (kidney, lung, endocrine, autoimmune diseases, pulmonary tuberculosis, and tumour) and had been routinely monitored (self-report or CDC-report) with HIV-1 test results every 3 months. WB and BED-CEIA were performed on all included samples.

### 2.2. WB Assay

WB for confirmation of HIV-1/2 was conducted with a commercial kit (HIV blot 2.2, MP Diagnostics, Singapore) according to the manufacturer's instructions. Interpretation of the WB bands was processed after the nitrocellulose membranes were naturally dry. The appearance of specific WB bands was determined visually by three professionals.

### 2.3. Quantitative Analysis of Grey Ratios on WB Bands

AlphaView Software was used to analyse the grey ratios of the WB bands in the section for multiple band analysis. The area of interest was manually drawn with a rectangle circled around the bands. The areas of the objects and background were selected according to the instruction. Given the different background levels across the WB profiles, multiregional background correction was adopted to subtract backgrounds accounting for each band to increase the accuracy of the densitometry analysis of the images. For each sample, serum control band was used as internal control band. The relative grey ratio of a targeted band was calculated as follows: background-corrected (BC) average of a targeted band/BC average of internal control band. BC average represents the region average after background subtraction.

### 2.4. BED-CEIA

The BED-CEIA was performed according to the manufacturer's instructions (Calypte Biomedical, Lake Oswego, OR). Samples were defined as recent infection if the OD-n in confirmation test was less than or equal to 0.8, otherwise as older infection.

### 2.5. Statistical Analysis

SPSS19.0 was used to conduct the Mann–Whitney test, Pearson chi-square test, and stepwise logistic regression analysis. The predicted probability was used to construct the receiver operating characteristic (ROC) curve. MedCalc (version 11.4.2.0; MedCalc, Mariakerke, Belgium) software was utilised to perform ROC analysis. A *p* value less than 0.05 was considered significant.

### 2.6. Ethical Statement

This study was approved by the Guangxi Institutional Review Board (GXIRB) with reference Number GXIRB2014-0041. All the participants were given full explanation and understood well this study. Written informed consent was signed by all participants.

## 3. Results

### 3.1. Characteristics of the Patients

Of the 81 HIV-infected individuals included in the training set, 43 were recent infections and 38 were older infections. Meanwhile, 72 eligible patients were enrolled in the validation set, and 26 of them were recent infections and 46 were older infections. The mean EDIs of recently infected patients in the two sets were 184 days and 227 days, respectively. For older infection, they were 1699 days and 1360 days, respectively. Overall, 90.85% of the eligible participants were male, similar to that in a previous study [15]. Ages, antiretroviral treatment, and CD4^+^ T cell counts were similar in both sets.

### 3.2. Frequency of HIV WB Bands

The WB band patterns in the training set were similar to that of the validation set.* Env* proteins, gp41, gp120, and gp160, were observed in all subjects. The most frequently lost bands were p39 and p55 in both recently infected and older infected patients. A significant difference of p55 frequency was observed between the recent infection and older infection groups (*p* < 0.05). The differences of the remaining band frequencies were nonsignificant (*p* > 0.05).

### 3.3. Relative Grey Ratio of WB Bands

**Figure 1:**
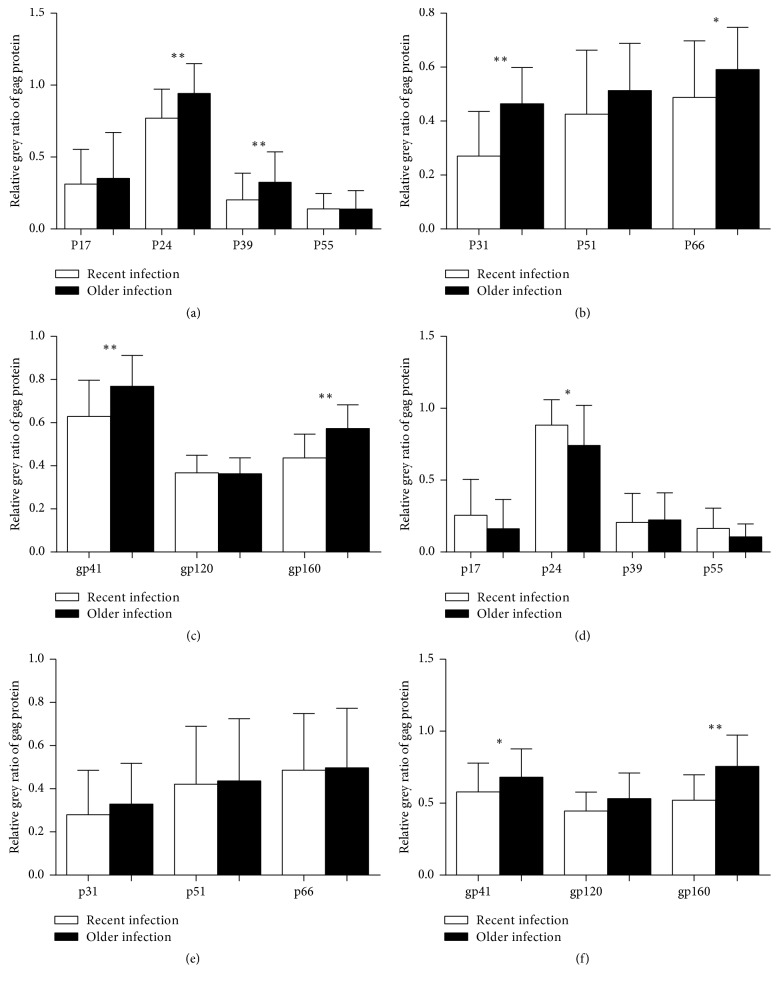
Relative grey ratio of WB bands in recent infection and older infection group. WB bands were analysed using software AlphaView. Serum control of each sample was utilised as internal control of the corresponding sample. Normalized grey value was compared by the Mann–Whitney test. The bar graph was visualised by GraphPad Prism 5.0. (a–c) Normalized grey value of multiple WB bands in training set. (d–f) Normalized grey value of multiple WB bands in validation set (^*∗*^*p* < 0.05; ^*∗∗*^*p* < 0.01).

The relative grey ratio of WB bands displayed a similar pattern in the training and validation sets except for p17 and p24. The results of* gag* protein,* pol* protein, and* env* protein of the training set are shown in Figures 1(a)–1(c) and those of the validation set in Figures 1(d)–1(f). The relative grey ratios of p24 and p39 were significantly different between recent HIV-1 infection and older infection in the training set (*p* < 0.01). For relative grey ratio of protein bands in the* pol* region, significant differences of p31 and p66 were also only observed in the training set between the two groups (*p* < 0.05, Figure 1(b)). The difference of gp41 and gp160 was significant in both training and validation sets (Figures 1(c) and 1(f)). In total, 6 of the 10 WB bands were different among individuals who had different EDIs in the training set, and two bands were observed in each gene region of HIV-1.

### 3.4. The Predictive WB Band Panel and Its Performance in Validation Set

We conducted univariate logistic regression analysis on the WB-based data, including present status and relative grey ratio of WB bands in the training set. The significant variables were further analysed after standardization. Collectively, relative grey ratios of p24, p39, p31, p66, gp41, and gp160 and the present status of p55 were further processed using multivariate logistic regression. Among the seven differential markers, four were stepwise included into the model by MedCalc software, as well as by SPSS19.0. A predicted model was then established on the basis of the following significant predictors:

(1) Logitz=−0.731−1.098×X1−1.477×X2−2.117×X3−2.822×X4.

*X*1 represented the standardized present status of p55. *X*1 is equal to zero in the presence of p55 and one otherwise. *X*2, *X*3, and *X*4 represented the standardized relative grey ratio of bands p31, gp41, and gp160, respectively. The probability of predicting recent HIV-1 infection was calculated as follows: *p* = *e*^*z*^/(1 + *e*^*z*^). The overall model fit was significant (*χ*^2^ = 65.760, *p* < 0.001).

**Figure 2:**
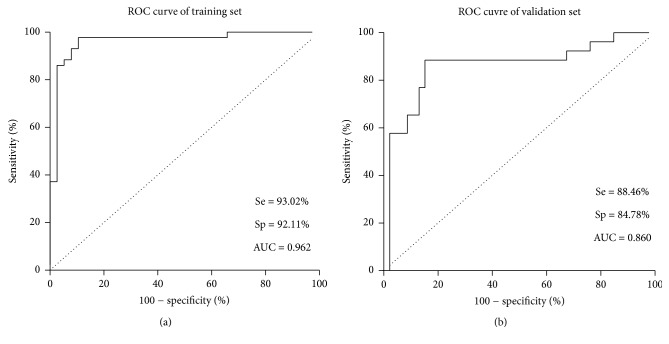
ROC analysis of WB-based model to identify recent HIV-1 infection. (a) WB method in the training set; (b) WB method in the validation set.

ROC analysis was subsequently conducted to evaluate the performance of the established model. The AUC was 0.962, with 95% confidence interval of 0.918 to 1.006. A *p* value of 0.487 was identified as the optimal cut-off point by ROC analysis, meaning that individuals with predicted *p* > 0.487 were classified as recent infection, otherwise, as older infection. Sensitivity and specificity of the predicted probability in training set were 93.02% and 92.11%, with an overall accuracy of 92.59% (Kappa = 0.851, Figure 2(a)). The performance of univariate ROC analysis on the significant predictors was also evaluated. The AUC, as well as sensitivity and specificity, was much lower than that of the multivariate results. The sensitivity, specificity, and overall agreement rate of the established model in validation set were 88.46%, 84.78%, and 86.11% (Kappa = 0.709), respectively, Figure 2(b).

For the evaluation of the effect of potential factors on the model, accuracy was calculated in subjects with different gender, age, treatment, CD4^+^ T cell counts, and infection routes. The accuracies of these factors were not significantly different among groups with different gender, age, treatment, CD4^+^ T cell counts, and infection route. This finding indicated that the model was not affected by these factors.

### 3.5. Performance of BED-CEIA

**Figure 3:**
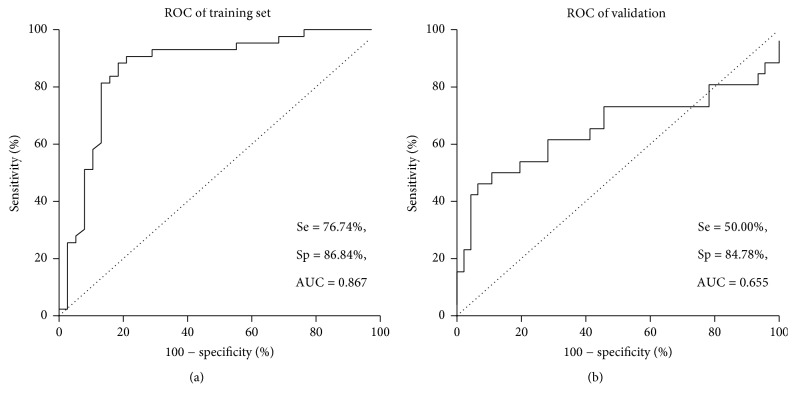
ROC analysis of BED-CEIA method to identify recent HIV-1 infection. (a) BED-CEIA method in the training set; (b) BED-CEIA method in the validation set.

BED-CEIA was performed in both sets with a referenced OD-n cut-off value of 0.8. The overall accuracy, sensitivity, and specificity in training set were 76.74%, 86.84%, and 81.48% (Kappa = 0.631), respectively (Figure 3(a)). In the validation set, the sensitivity and specificity were 50.00%, 84.78%, and 72.22% (Kappa = 0.366), respectively (Figure 3(b)).

### 3.6. Comparison of WB-Based Model and BED-CEIA Method

**Figure 4:**
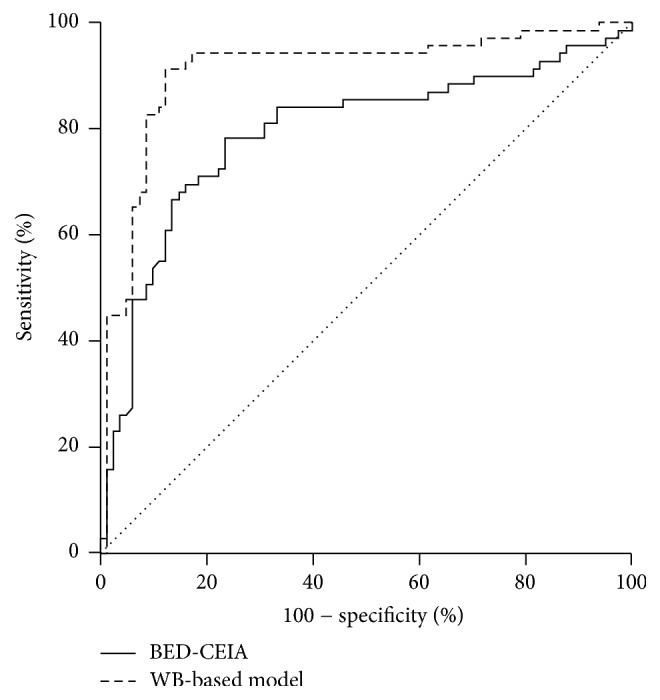
Comparison of ROCs of WB-based model and BED-CEIA method. — represents the ROC curve of BED-CEIA method; – – – represents the ROC curve of WB method.

The overall performance of WB-based model was found to be better than BED-CEIA after their ROC results were compared (Figure 4, *p* < 0.05). Furthermore, a total of 39.22% participants were classified as recent infections by BED-CEIA, while 47.71% were classified by the WB-based model (Table 1). Among the participants who were infected for less than or equal to 0.42 years (153 days), 61.90% and 85.71% individuals were correctly identified by BED-CEIA and WB-based models, respectively. For individuals infected for 0.42 years to 1 year, the WB-based model has a higher accuracy than that of BED-CEIA (*p* < 0.05). Among the samples collected from individuals infected for more than 1 year, the proportions of samples classified by BED-CEIA as indicative of recent infections were similar to that of WB-based model (*p* > 0.05). Overall, by using a recency period of 1 year, the WB-based model was able to correctly classify 91.30% as recent infection, which was higher than that of BED-CEIA (66.67%, *p* < 0.05).

## 4. Discussion

The purpose of this study is to establish a simple and convenient method to determine new HIV infections on the basis of the WB strips data collected in the previous years to evaluate the effectiveness of AIDS prevention and control. Considering the actual work in China (government estimated the AIDS epidemic at the end of every single year to guide the following year's work), but also referring to other related studies [2, 16, 17], we used 1 year as a threshold to clarify recent and older infection. Meanwhile, as only patients with relatively accurate infection time were enrolled, the sample size of the participants in this study was limited.

The appearance of all bands in both recent infection and older infection of HIV-1 in this study was frequently observed as the major protein transcribed by* env*,* gag*, and* pol* formed rapidly within 4 months of infection during the development of the immune response to HIV-1 [12, 15, 18]. Although specific band patterns were found by Schüpbach et al. [3], we failed to identify the same patterns in our study where almost all the frequencies of WB bands were similar between the two groups. Nevertheless, we did find a consistent pattern that p55 was the most frequently missing band, as previously reported by other studies [19]. The lower present frequency of p55 in recent infection may be due to the EDIs that were insufficiently long for p55 maturation, as it would present persistently once it appeared. Lange et al. also revealed that seroconversion antibodies to* env* antigens have been detected in all virtual HIV-infected persons regardless of clinical stage [20]. In our study, we observed a similar pattern that all the participants presented three* env*-antibodies despite the EDIs. Another study showed that lack of p31 was considered as a predictor of seroconversion, and the presence of all* env* glycoproteins, with p24 and/or p51, was used to predict late-stage HIV-1 infection [11]; this pattern was, however, not noted in this study. The reason could be that the previous study included all available HIV-1-positive individuals and grouped them by WHO stages. We followed strict criteria for participant enrolment. More specifically, we grouped the participants according to their estimated infection duration, which provides more accurate information of time-dependent evolution of antibodies. On the other hand, the included early infections in this study might not be sufficient because these antibodies take usually 2 to 4 months to appear [21]. Overall, the WB band pattern-based method for identification of recent HIV-1 was reported to be unstable by the above-mentioned studies. To explore a more stable and reliable classified algorithm than the band pattern-based method, we included the normalized relative expression levels of the 10 HIV-1 antibodies based on previous studies [12, 22]. Six of the WB bands were significantly higher in older infections in the training set, involving antibodies from three major regions of HIV-1 (*env*,* pol*, and* gag*). The results suggested that the responses of the antibodies persistently evolved as EDI changed [22, 23] rather than only during the first several months of infection [12]. The low sensitivity and specificity of single-factor algorithms in recent HIV-1 infection diagnosis suggest that multiple markers should be taken into consideration to achieve a better performance [24].

As the stepwise logistic regression model has shown its potential on disease diagnosis using multiple variables [25], we formulated a similar model using the seven markers that exhibited significant difference in training set. As a result, only markers that showed the same tendency in both training and validation sets were stepwise included into the model. The established model was found to present better sensitivity and specificity in both sets of this study than those of BED-CEIA method and univariate algorithm [3, 11]. The absence or lower expression levels of the predictors suggested a lower risk of being diagnosed as a recent infection. Meanwhile, their presence or higher expression levels may contribute to predicting older infection. gp41- and p31-specific antibodies appeared earlier during acute HIV-1 infection, making them capable of predicting HIV-1 infection in the early stage [12]. Furthermore, lack of p31-specific antibody was found to be significantly associated with early HIV-1 infection [19]. In addition,* env* protein plays an important role in HIV-1 entrance by providing binding site for CD4^+^ receptor and coreceptors. Therefore, having two* env* proteins possessing the potential to be predictors of recent infection was comprehensible. A previous study conducted in China found that “recent” infections showed lower mean antibody intensities to these HIV protein antibodies in WB when compared to those established infections [14]. We further developed this advance approach by means of a logistic regression model, based on their grey value as shown on WB bands, which already demonstrated high sensitivity and specificity in the determination of recent HIV-1 infection.

Though a number of studies have revealed its overestimation of recent HIV-1 infections [4, 26], BED-CEIA is still the most widely used method in China. BED-CEIA was developed for HIV surveillance; thus in this study we used it as a control method to access our new approach, in terms of its recency period; a similar study was conducted in Korea [27]. In this study, we addressed evidence that the performance of BED-CEIA to accurately estimate the recent HIV-1 infection was ineffective. The sensitivity and specificity in both sets were lower than those of the WB-based model. Among participants infected for 0.42 years to 1 year, the WB-based model has a higher accuracy than that of BED-CEIA. Even in participants who were infected within 0.42 years, which is the recency period value of recent infection by BED-CEIA, the method had a very low accuracy in identifying those who were actually infected within 0.42 years. Overall, by using a recency period of 1 year, the WB-based model was able to correctly classify 91.30% as recent infection, which is defined by an EDI of 1 year. Besides, BED-CEIA was significantly influenced by antiretroviral treatment shown in other studies [4, 26]. Given that multiple markers were recommended [22, 24, 28], we included several markers that stably showed distinction between recent infection and older infection, while BED-CEIA relies completely on optical density reading of gp41-specific IgG proportion in blood [29]. The multiple markers in the WB-based model made it less likely to be affected by ART, showing a similar high accuracy in treated and untreated populations. On the other hand, considering that the band intensity of the antibodies depends strongly on the plasma concentration of HIV-1 RNA, which was influenced by ART [30], we set serum control band as an internal control to improve the comparability of each strip and different test batches in WB and thus improve the accuracy of our model.

Rather than developing a diagnostic strategy for HIV-1 acute infection, this study aimed to explore an available method from the practical settings to compare the practicability of WB-based method and BED-CEIA and not the value of BED-CEIA itself as a diagnostic strategy. The enrolment of the study population was also based on the actual work.

## 5. Conclusion

In this study, a binary stepwise logistic regression model was established based on a panel of variables to determine recent HIV-1 infections in a recency period of 1 year. The model can better differentiate recent HIV-1 infection from older infection than BED-CEIA and was not affected by gender, age, ART, and CD4^+^ T cell counts. The method can be potentially used for the routine estimation of HIV-1 incidence and prediction of AIDS epidemic, especially in resource-limited regions where new-generation assays remain limitedly accessible.

## Figures and Tables

**Table 1:** Samples classified as recent infections by BED-CEIA and WB-based model.

EDI (Y)	Sample tested (number)	Samples classified as recent infections (number; %)		*p*
		BED-CEIA, 153 d	WB-based model, 365 d	
0.00 to 0.42	21	13 (61.90)	18 (85.71)	0.079
0.42 to 1	48	34 (70.83)	45 (93.75)	0.003
>1.00	84	13 (15.48)	10 (11.90)	0.501

Total	153	60 (39.22)	73 (47.71)	0.134
